# Bailout Procedure Utilizing Balloon Dilatation for a Percutaneous Micro-axial Flow Pump Entrapped Within a Significantly Calcified Subclavian Artery

**DOI:** 10.7759/cureus.65804

**Published:** 2024-07-30

**Authors:** Kazuyoshi Takagi, Hiroyuki Otsuka, Kosuke Saku, Eiki Tayama

**Affiliations:** 1 Division of Cardiovascular Surgery, Department of Surgery, Kurume University, Kurume, JPN

**Keywords:** subclavian calcification, vascular complication, balloon dilatation, endovascular angioplasty, impella device

## Abstract

The IMPELLA 5.5 (Abiomed Inc., Danvers, Massachusetts, United States) is a catheter-based, micro-axial blood pump designed to enhance organ perfusion in patients with cardiogenic shock. Despite its superior hemodynamic support, vascular complications are a significant concern, with many patients needing to discontinue IMPELLA therapy due to these issues. Patients may even require surgical intervention to address device-related vascular injuries. The IMPELLA 5.5 implantation in vessels with severe calcification is particularly associated with complications such as vascular calcification, stenosis, vascular tortuosity, and the use of larger sheaths are risk factors following endovascular therapy and IMPELLA implantation. In this report, we present a case of severe calcification in the right subclavian artery, in which the IMPELLA 5.5 was lodged. The calcifications protruded into the vascular lumen, becoming lodged between the IMPELLA motor and the cannula, complicating extraction despite the vessel having sufficient diameter. We successfully removed the device using a balloon dilation technique, ensuring safe extraction. No vascular complications such as pseudoaneurysm or dissection were observed in the right subclavian artery one month after extraction. This case highlights a potential approach for managing similar complications and vascular access for IMPELLA insertion.

## Introduction

The IMPELLA (Abiomed Inc., Danvers, Massachusetts, United States) is a catheter-based, micro-axial blood pump engineered to enhance organ perfusion by increasing mean arterial pressure, reducing left ventricular volume and pressure, and lowering myocardial oxygen consumption. These combined effects are expected to improve the prognosis of patients with cardiogenic shock, leading to its widespread and rapidly growing use [1]. The IMPELLA 5.5 model (IM5.5), capable of providing a flow rate of up to 5.5 L/minute, offers comprehensive ventricular support with superior hemodynamic stability over extended periods. It is highly effective not only as a bridge to recovery but also as a bridge to durable ventricular assist devices or heart transplantation [2,3]. One notable advantage of the IM5.5 is its capability for axillary placement, which facilitates patient mobilization and rehabilitation [2].

However, vascular complications (VCs) are a significant concern with IMPELLA implantation, reported in 3-17% of cases [1,4,5]. Among these, vascular perforation and rupture are particularly concerning as they can be fatal and may result in limb loss or mortality. This issue is particularly challenging with the IM5.5, which necessitates access vessels of at least 7 mm in diameter [2]. Consequently, meticulous monitoring for VCs is crucial when utilizing the IM5.5.

Several reports have revealed the effectiveness of covered stent in managing iatrogenic artery perforation and pseudoaneurysm associated with IMPELLA extraction [6,7]. However, there are no reports of preventive concomitant endovascular therapy (EVT) preventing VCs during IMPELLA extraction. In this report, we present a case where an IM5.5 device became entrapped in severe calcification of the right subclavian artery. Standard removal was inhibited due to the advanced calcification of the access vessels. Nevertheless, we successfully removed the device using a balloon dilation technique, ensuring safe extraction and highlighting a potential approach for similar complications.

## Case presentation

**Figure 1:**
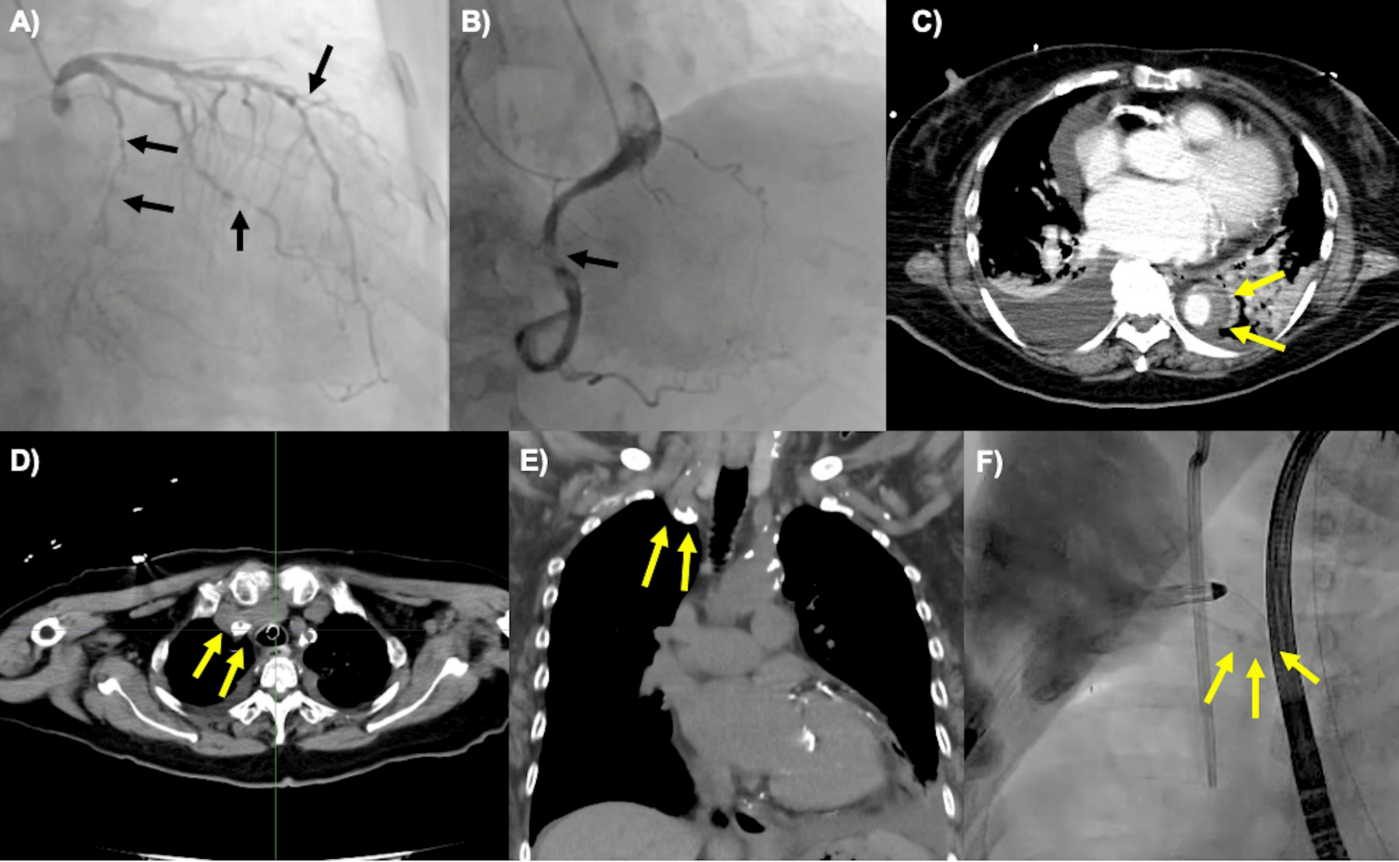
Preoperative CAG, CT, and intraoperative findings during IMPELLA 5.5 insertion (A, B) CAG shows significant stenosis. The black arrows indicate 75% stenosis of the left anterior descending artery, 75-90% stenosis of the left circumflex coronary artery, and 90% stenosis of the right coronary artery. (C) CT reveals a thrombosed type B aortic dissection (yellow arrows). (D, E, F) CT and intraoperative angiography demonstrate severe calcification of the right subclavian artery, as indicated by the yellow arrows. The calcification extended into the vascular lumen (E). CAG: coronary angiography IMPELLA 5.5; Abiomed Inc., Danvers, Massachusetts, United States

A 77-year-old female with a history of type 2 diabetes mellitus and previous urgent admission for heart failure one year ago was diagnosed with arteriosclerosis obliterans (ASO) and subsequently underwent EVT. Because preoperative tests for EVT indicated reduced left ventricular function, this prompted concurrent coronary angiography which revealed significant stenosis in three coronary arteries: 75% stenosis in the midsection of the left anterior descending artery, 90% stenosis in the midsection of the left circumflex artery, and 90% stenosis of the right coronary artery (Figure 1A, 1B). Based on these findings, percutaneous coronary intervention or coronary artery bypass grafting (CABG) was recommended.

Two weeks later, the patient presented to the hospital with symptoms of dyspnea and leg edema. During her evaluation in the emergency room, she experienced cardiopulmonary arrest with an initial rhythm of pulseless electrical activity. Cardiopulmonary resuscitation (CPR) was promptly initiated, and spontaneous circulation was achieved after 10 minutes. An electrocardiogram showed negative T waves in leads V3-6, and serum levels of creatinine kinase (CK) and CK-myocardial band (MB) were mildly elevated at 316 IU/L and 39 IU/L, respectively. An echocardiogram revealed severely reduced cardiac function with a left ventricular ejection fraction of 10-20%. A contrast-enhanced computed tomography (CT) scan identified an acute Stanford type B aortic dissection with thrombus occlusion (Figure 1C).

Although systolic blood pressure was maintained at 101 mmHg with 0.04 μg/ml/min of norepinephrine support, mechanical circulatory assist was deemed necessary to prevent further deterioration of circulation following cardiopulmonary arrest in the context of ischemic heart disease and significantly reduced left ventricular function. Type B aortic dissection and ASO made device placement from the femoral artery difficult, and the need for long-term support post coronary revascularization further complicated the situation. Therefore, IM5.5 implantation from the right axillary artery was deemed optimal. The right axillary artery, with an overall vessel diameter of at least 7 mm, was suitable for IM5.5 placement under general anesthesia. However, advancing the IM5.5 proved challenging due to significant calcification at the bifurcation with the common carotid artery (Figure 1D, 1E, 1F). Despite these difficulties, the IM5.5 was successfully positioned in the left ventricle (Video 1).

**Video 1:** Insertion and extraction of IMPELLA 5.5. IMPELLA 5.5; Abiomed Inc., Danvers, Massachusetts, United States

To allow for the patient’s renal function recovery and to administer hypothermia following CPR, CABG was scheduled for the fourth day after IMPELLA implantation. The patient underwent heart-beating CABG under the support of the IM5.5 without complications. Three days post CABG, her hemodynamic stability allowed for the weaning off of the IMPELLA.

**Figure 2:**
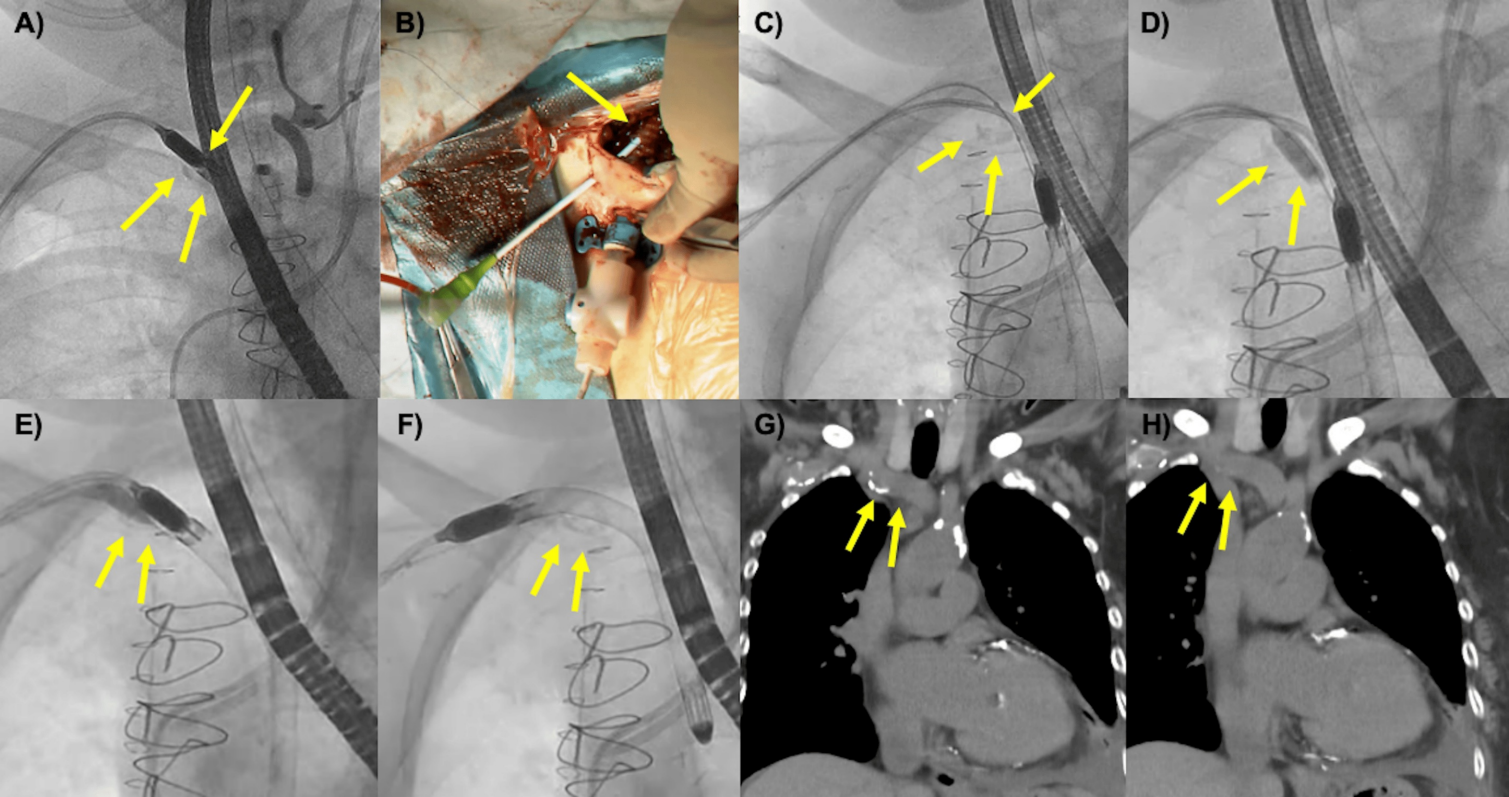
Intraoperative findings during the extraction of IMPELLA 5.5 (A) The yellow arrow points to the IMPELLA 5.5 device trapped in the calcified area of the subclavian artery. The calcification lodged into the space between the motor and the cannula. (B) The yellow arrow indicates the placement of a 6 Fr sheath adjacent to the graft. (C, D) The 7 mm x 40 cm balloon dilatation catheter was positioned at the calcified region. Initial dilations were performed at a pressure of 10 atm. (E) Slow deflation of the balloon with the IMPELLA 5.5 positioned on it, facilitating the device's passage through the calcified area. (F) The IMPELLA 5.5 was successfully removed after passing through the calcification. (G, H) No vascular complication was detected one month after the extraction of IMPELLA 5.5. IMPELLA 5.5; Abiomed Inc., Danvers, Massachusetts, United States

However, attempts to remove the IMPELLA using standard techniques were unsuccessful due to its entrapment in the calcified area of the right subclavian artery. The calcifications that protruded into the vascular lumen were lodged in the space between the IMPELLA motor and the cannula. When the IM5.5 was pulled, the vessel was significantly pulled along with the calcification, posing a high risk of vascular injury (Figure 2A) (Video 1). To address this, the right subclavian wound was reopened. A tobacco-pouch suture was placed on the graft using a 5-0 polyvinylidene fluoride suture, and a 6 Fr sheath was inserted (Figure 2B). A 7 mm x 40 cm Mustang Balloon Dilatation Catheter (Boston Scientific Corporation, Marlborough, Massachusetts, United States) was advanced to the calcified region (Figure 2C). Initial dilations at 10 atm pressure for 30 seconds, performed twice to achieve a 7 mm diameter as per the instruction for use, were insufficient for extraction (Figure 2D). Further dilations at 12 atm pressure for 30 seconds to achieve a 7.2 mm diameter were then carried out. With the IM5.5 positioned on the balloon, careful and slow deflation allowed the device to pass through the calcification and be successfully removed (Figure 2E, 2F) (Video 1). One month post the extraction of IM5.5, plain CT imaging showed no VCs such as pseudoaneurysm or dissection in the right subclavian artery (Figure 2G, 2H). Postoperative echocardiography revealed an improved left ventricular ejection fraction of 50-60%, and the patient was discharged without major complications. 

## Discussion

Overview of VCs

VCs, including hematoma, acute limb ischemia, vascular injury, and pseudoaneurysm, are frequently reported following IMPELLA implantation [1,4,5]. Among these, vascular perforation and rupture are particularly concerning as they can be fatal and may result in limb loss or mortality. In a review by Abaunza et al., VCs were found to occur in 1-17% of cases, with approximately one-third of these patients needing to discontinue IMPELLA therapy due to these issues [4]. Additional studies indicate that about 4-5% of patients required surgical intervention to address vascular injuries related to the device [5,8]. These severe complications highlight the critical need for careful monitoring and management during the use of IMPELLA systems.

Risk factors of VCs

Several risk factors contribute to the development of VCs following EVT and IMPELLA implantation. These factors include vascular calcification, stenosis, vascular tortuosity, female sex, older age, low body surface area, chronic kidney disease, emergency procedures, steroid use, diabetes mellitus, and the use of larger sheaths [4,9,10]. Notably, every 1 mm decrease in vessel diameter is associated with a 35% increase in the risk of VCs following IMPELLA insertion [4]. For the safe implantation of IM5.5, it is essential to evaluate not only the site of graft anastomosis but also the entire vessel diameter, the presence of calcification or stenosis, and any associated risk factors. 

**Figure 3:**
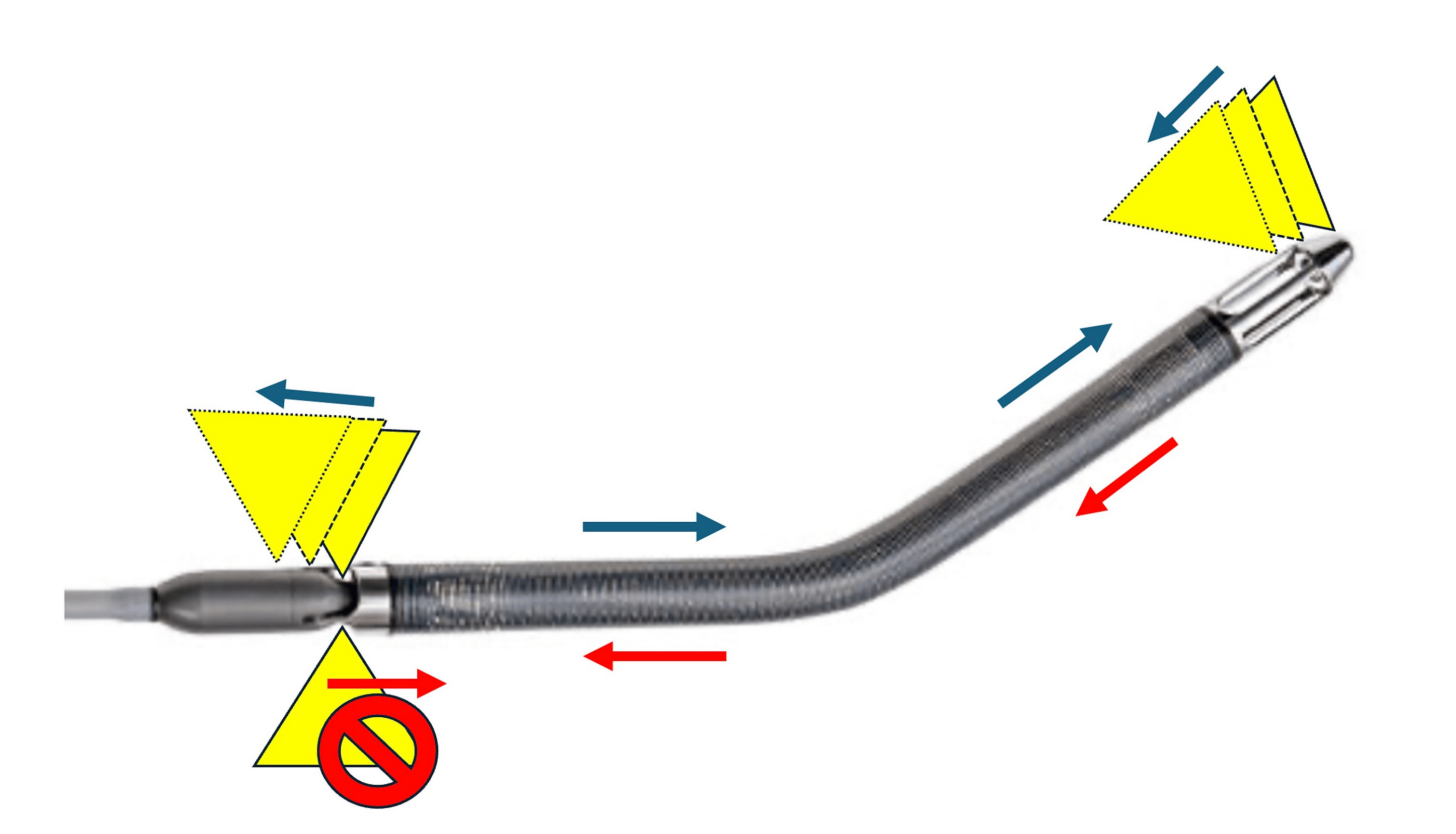
Mechanism by which calcification was lodged in the space between the IMPELLA motor and the cannula during extraction. During insertion, the calcification passes through due to the dilator-like effect of the IMPELLA 5.5 itself. However, during removal, the calcification tends to lodge into the gap between the motor and the cannula. Yellow triangles: The calcifications protruding into the vascular lumen. Blue arrows: The direction of movement of the IMPELLA 5.5 and calcification during IMPELLA insertion. Red arrows: The direction of movement of the IMPELLA 5.5 and calcification during IMPELLA extraction. IMPELLA 5.5; Abiomed Inc., Danvers, Massachusetts, United States

The IM5.5 design acts as a dilator during insertion, which allows it to be placed in vessels slightly smaller than 7 mm or those with local calcification or stenosis (Figure 3). As demonstrated in this case, even with an overall vessel diameter of 7 mm, localized calcification can make insertion challenging, because vessels with calcified lesions do not easily expand and calcification itself is trapped in the device. If the use of IM5.5 is absolutely necessary, passing a 21 Fr sheath through the calcified site to confirm passage prior to insertion may be useful, but if this is not feasible, an alternative approach should be considered [11,12].

Additionally, the design of the IMPELLA device itself presents a unique risk; the space between the IMPELLA motor and the cannula is prone to capturing calcifications that extend into the vascular lumen. If these calcifications become lodged during the extraction process, they can be particularly difficult to dislodge, increasing the risk of VCs (Figure 3).

Case analysis

In the presented case, the patient exhibited multiple risk factors, including severe calcification that extended into the subclavian artery, female sex, diabetes mellitus, and the need for a large-diameter device. These factors collectively heightened the patient's risk of developing VCs. 

To minimize these risks during IMPELLA removal, we utilized fluoroscopic guidance. This technique provided precise visualization and enabled careful manipulation of the device, reducing the potential for vascular injury. In addition, fluoroscopy can help avoid unforeseen VCs and resolve issues with concurrent EVT. The use of fluoroscopic guidance facilitated the safe and effective extraction of the IMPELLA device, demonstrating its value in managing complex cases with multiple risk factors for VCs. Therefore, we recommend using fluoroscopy in all cases involving the removal of an IM5.5 with calcified, stenotic, or tortuous vessels and bends that complicate insertion. Even with fluoroscopy, forced removal should be avoided. If removal proves difficult, the patient should be transitioned to concurrent EVT or surgical removal, as shown in this case.

The severe calcification of the subclavian artery posed significant challenges during IMPELLA implantation. Anticipating potential vascular injury during removal, we prepared for balloon dilation under fluoroscopic guidance. In this case, a 7 mm balloon was selected to match the diameter of the IM5.5. Although the prophylactic use of balloon dilation proved effective, allowing for the safe and uncomplicated removal of the IMPELLA device, it was difficult to pass with nominal pressure dilation. Perhaps a slightly larger balloon should be selected for balloon dilation of calcified lesions. 

EVT for calcification, however, requires specific precautions. Dilation is more likely to be effective in cases of circumferential calcification. In cases of ubiquitous calcification, altering positioning and using an intervening balloon to release calcified segments might be beneficial.

This case is notable as the first reported instance, to the best of our knowledge, where prophylactic EVT was successfully used to facilitate IMPELLA removal. This preemptive measure allowed for the safe and uncomplicated removal of the IMPELLA device, highlighting its potential for managing complex cases with severe vascular calcification. 

Implications and future directions

In the context of the transaxillary approach, establishing a sheath adjacent to the graft and performing EVT can be effective in reducing bleeding from the sheath entry site. This technique may also assist in the placement of IMPELLA devices, especially when using larger devices or when dealing with compromised access vessels. Performing EVT prior to the insertion of large IMPELLA devices such as the IM5.5 could be particularly beneficial in ensuring smoother device implantation and managing potential VCs. In this case, pre-implantation EVT might have simplified both implantation and removal. However, careful consideration of stent size is crucial, as improperly sized stents can complicate the passage of the IM5.5. 

Moreover, through assessment of vascular access and proactive pre- and post-IMPELLA implantation interventions by the vascular team are essential. These measures can enhance the safety and effectiveness of using the IMPELLA device and potentially expand the range of cases where the device can be safely used.

Previous reports

Many reports have documented the effectiveness of EVT in managing VCs associated with IMPELLA extraction [6,7]. For example, Awan et al. described the successful use of a covered stent to treat an iatrogenic perforation of the external iliac artery during IMPELLA removal [6]. Another case report highlighted the use of a covered stent to manage a ruptured iatrogenic pseudoaneurysm of the subclavian artery that developed six weeks after the removal of a transaxillary IMPELLA device [7].

## Conclusions

In this report, we described a challenging case involving severe calcification of the subclavian artery that complicated the removal of an IMPELLA 5.5 device. Despite the vessel having a sufficient diameter, calcifications protruding into the vascular lumen became lodged between the IMPELLA motor and the cannula, complicating extraction. We successfully managed this complication using a balloon dilation technique, which facilitated the safe removal of the device. This case highlights the critical issue of vascular complications associated with IMPELLA insertion and removal. Such complications can lead to severe outcomes, including discontinuation of IMPELLA support, limb loss, or life-threatening conditions. To mitigate these risks, it is essential to thoroughly understand the risk factors, such as vascular calcification and stenosis, and to meticulously prepare for both implantation and removal procedures.

The successful application of prophylactic EVT in this case underscores the importance of an interdisciplinary approach that includes both the heart team and vascular specialists. This strategy not only improves procedural success but also enhances patient outcomes by addressing potential complications proactively. Incorporating EVT prior to device implantation and during removal can significantly reduce the risk of vascular complications. Our findings contribute to the existing body of knowledge by demonstrating the effectiveness of balloon dilation and prophylactic EVT in managing complex cases with severe calcification. This case serves as a valuable example of how these techniques can be applied to improve procedural safety and patient outcomes. Future research should investigate the long-term outcomes of EVT for IMPELLA-related vascular complications and explore other innovative techniques to enhance device management. Emphasizing patient-centered care, these strategies not only improve the immediate success of the procedure but also enhance overall patient quality of life. By integrating these practices into clinical guidelines, healthcare providers can better manage the complexities of IMPELLA support and improve patient safety.
